# Regulating charge transfer of copper(i) coordination compounds *via* conformation engineering for highly efficient radioluminescence and 3D X-ray imaging

**DOI:** 10.1039/d5sc06329a

**Published:** 2025-10-22

**Authors:** Yongkang Zhu, Yongjing Deng, Qianxi Li, Ning Ding, Yulong Wang, Mengzhu Wang, Kenneth Yin Zhang, Shujuan Liu, Qiang Zhao

**Affiliations:** a State Key Laboratory of Flexible Electronics (LoFE), Institute of Advanced Materials (IAM) & Institute of Flexible Electronics (Future Technology), Nanjing University of Posts and Telecommunications Nanjing 210023 P. R. China iamsjliu@njupt.edu.cn; b School of Electronics and Information Technology, Nanjing University of Information Science and Technology Nanjing 210044 P. R. China iamqzhao@nuist.edu.cn

## Abstract

Copper(i) coordination compounds have emerged as promising candidates for X-ray scintillators because of their superior X-ray absorption capacity and tunable radioluminescence. However, a strategy that realizes efficient radioluminescence by varying the competition between radiative ligand-related transitions and nonradiative cluster-centered charge transfer is yet to be clearly demonstrated. Here, *via* conformation engineering, a series of copper(i) iodide coordination clusters have been designed and synthesized, and named (POPy)_4_Cu_2_I_2_, (POPy)_4_Cu_4_I_4_-α and (POPy)_4_Cu_4_I_4_-β (POPy = 4-phenoxypyridine). Mechanistic studies reveal that the radioluminescence in isomeric Cu_4_I_4_ clusters originates from triplet metal/halide-to-ligand charge transfer (^3^M/XLCT) and cluster-centered (^3^CC) excited states, respectively. The experimental results disclose that intramolecular charge transfer is desirable for radioluminescence as it can transfer excitons generated by absorbing radiation ionizations to ligands to form thermal electrons. Due to the large X-ray absorption cross-section of the Cu_4_I_4_ cube and efficient ^3^M/XLCT emissions, the scintillation film fabricated by using (POPy)_4_Cu_4_I_4_-α realizes a high resolution of 20.2 lp mm^−1^, and further enables a 3D X-ray imaging demonstration. Our work provides a comprehensive comparison of the radioluminescence characteristics between ^3^M/XLCT and ^3^CC excited states in isostructural Cu_4_I_4_-based clusters, providing a template for enhancing scintillation properties through conformation engineering.

## Abstract 2

A conformation engineering strategy regulates the charge transfer process of copper(i) halide clusters. The scintillation film fabricated by using the [Cu_4_I_4_]-based cluster delivers efficient radioluminescence and a 3D X-ray imaging demonstration.

## Graphical abstract

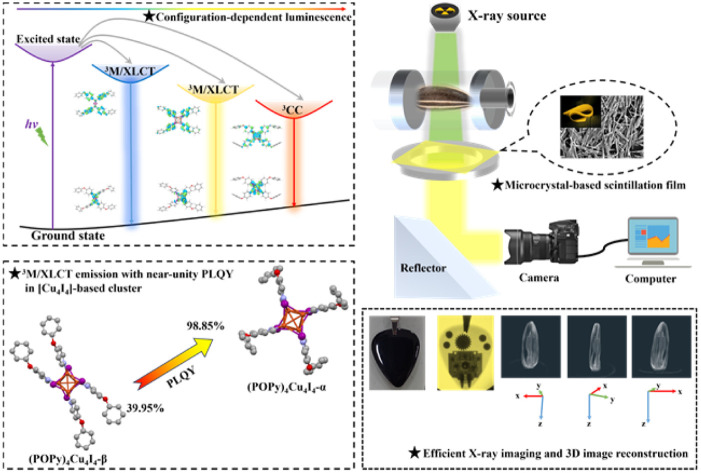

## Introduction

Scintillators, which emit visible light in response to high energy radiation like X-rays and gamma rays, play a pivotal role in the fields of industrial nondestructive testing, space security inspection, biomedical imaging and so on.^1–6^ The pursuit of new scintillators with superior performance, customizable emission colors and low costs is of great interest in materials chemistry.^7–9^ The reported CsPbX_3_ (X = Cl, Br or I) nanocrystal scintillators with efficient radioluminescence (RL) demonstrate the feasibility to tune radioluminescence covering the entire visible region, which indicates the great potential of metal halides for highly sensitive scintillators.^10^ Encouragingly, recent studies reveal that numerous lead-free metal halides can deliver scintillation performance rivaling or even surpassing that of lead-based scintillators.^11^ Therefore, developing lead-free materials with robust stability, low-cost fabrication and efficient radioluminescence to broaden the family of scintillators is of great significance.

**Scheme 1:**
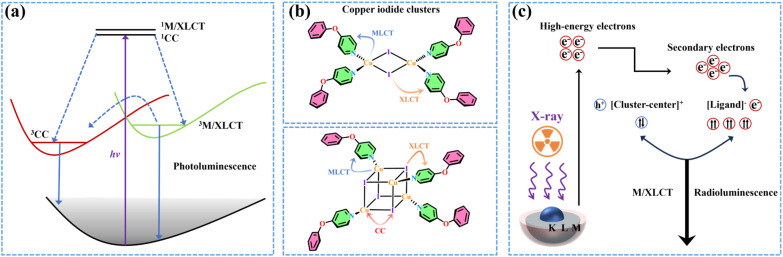
(a) Photophysical processes of copper iodide coordination clusters. (b) Transition characteristics of copper iodide coordination clusters. (c) Schematic diagram of the scintillation process in clusters.

Copper(i) coordination compounds possess several advantageous characteristics, including but not limited to cost-effectiveness, high chemical stability, excellent optical tunability and superior luminescence, thereby arousing significant research interest.^12–15^ In the family of copper(i) halide coordination clusters, inorganic modules and organic ligands are connected through Cu–N/P/S coordination bonds.^16^ Due to the various coordination environments, there are diverse forms of inorganic skeletons, such as monomeric [CuX], dimeric [Cu_2_X_2_], trimeric [Cu_3_X_3_], tetrameric [Cu_4_X_4_] and staircase-type [CuX]_*n*_ polymeric chains.^17–21^ In this case, the charge transfer pathways from the ground state to the excited state are complicated, which are associated with inorganic backbones and organic ligands. Typically, the charge-transfer excited states in these clusters can be attributed to several categories, such as the triplet cluster-centered (^3^CC) state, as well as the metal/halide-to-ligand charge transfer (^3^M/XLCT) state, also known as the intramolecular charge transfer state.^22^ The ^3^CC transition, including the metal-to-halide charge transfer process, has been found in numerous structures with strong Cu–Cu interactions, especially Cu_4_I_4_ cubes.^23^ However, the ^3^CC state is inferior to the other excited state, as it serves as the quenching site of excited energy and suppresses the radiative transition.^24^ Therefore, the clusters with ^3^CC emission are considered to be not suitable for efficiently luminescent applications. The ^3^M/XLCT state has been manifested to be highly effective in enhancing optical properties in these clusters.^25^ Under ultraviolet excitation, the ^3^M/XLCT and ^3^CC excited states in these clusters exhibit a competitive relationship, eventually resulting in the luminescent behavior in response to stimuli such as pressure and temperature variation.^26^ When ionized by X-rays, the clusters generate high-energy electrons that are ultimately captured by the organic ligands, producing radioluminescence dominated by intramolecular charge-transfer excited states (Scheme 1).

To gain efficient radioluminescence in copper(i) halide coordination clusters, it is very urgent to find effective strategies to achieve intramolecular charge-transfer predominant characteristics. Through ligand engineering, Xu *et al.* modified organic ligands with electron-donating groups, achieving intramolecular charge-transfer luminescence and efficient scintillation performance.^27^ Ju's group also achieved remarkable ligand-related radioluminescence by regulating the coordination mode of inorganic components.^28^ Intriguingly, some research studies have reported that different structures can be formed with the same chemical formula, which provides inspiration for regulating charge transfer processes by using isomers.^29,30^ However, there still remains a lack of conformation engineering strategies for realizing superb radioluminescence by regulating the ^3^M/XLCT and ^3^CC excited states in isostructural copper(i) halide coordination clusters.

Herein, we proposed a conformation engineering strategy to regulate the electron transition process. Taking 4-phenoxypyridine (POPy) as an organic ligand, a series of copper(i) iodide coordination clusters were synthesized by the liquid-phase diffusion method, and named (POPy)_4_Cu_2_I_2_, (POPy)_4_Cu_4_I_4_-α and (POPy)_4_Cu_4_I_4_-β, respectively. The conformational changes caused by the effects of flexible chains containing an O atom linker on the ligands are considered to be the key reason for the self-assembly of the isomeric [Cu_4_I_4_] clusters. Through the regulation of inorganic modules, (POPy)_4_Cu_2_I_2_, (POPy)_4_Cu_4_I_4_-α and (POPy)_4_Cu_4_I_4_-β exhibit blue, yellow and red emission respectively, under both ultraviolet and X-ray irradiation, almost across the entire visible spectrum. Experiments and density functional theory (DFT) calculations indicate that the emissions of (POPy)_4_Cu_4_I_4_-α and (POPy)_4_Cu_4_I_4_-β originate from ^3^M/XLCT and ^3^CC excited states, respectively, which is attributed to the different charge distributions caused by the conformational changes. Benefiting from the large X-ray absorption cross section of the [Cu_4_I_4_] core and efficient ^3^M/XLCT emission, (POPy)_4_Cu_4_I_4_-α achieves excellent scintillation properties, including a high light yield of 36 700 photons per MeV and a low detection limit of 97.63 nGy_air_ s^−1^. Furthermore, the flexible scintillation film fabricated by using (POPy)_4_Cu_4_I_4_-α microcrystal inks achieves a high spatial resolution of 20.2 lp mm^−1^ and three-dimensional (3D) X-ray imaging. This work not only provides a superb template for understanding the structure–property relationship of the isomeric copper(i) halide clusters, but also demonstrates their great potential as high-performance scintillators.

## Results and discussion

### Synthesis and structural characterization

**Fig. 1:**
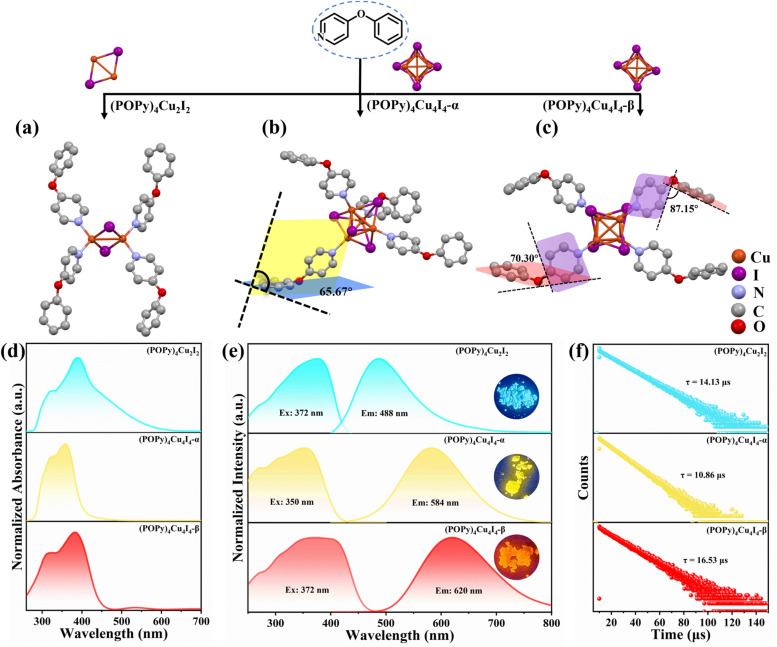
Crystal structures of (a) (POPy)_4_Cu_2_I_2_, (b) (POPy)_4_Cu_4_I_4_-α and (c) (POPy)_4_Cu_4_I_4_-β. All hydrogen atoms have been omitted for clarity. (d) Normalized UV-visible absorption spectra of the obtained complexes. (e) Normalized excitation and emission spectra of the obtained complexes. (f) Decay lifetime of the obtained complexes.

Crystals of the target compounds were prepared by the liquid-phase diffusion method (see the Experimental section). In brief, when the molar ratio of POPy to CuI is 2 : 1, or POPy is in excess, (POPy)_4_Cu_2_I_2_ crystals are preferentially formed. (POPy)_4_Cu_4_I_4_-α crystals are readily obtained by rapid diffusion from equimolar (1 : 1) POPy and CuI. The synthesis of (POPy)_4_Cu_4_I_4_-β crystals likewise adopts an equimolar feed ratio, but requires a slower, more precisely controlled crystallization rate than the former. When acetone is employed as the solvent for POPy, crystallization is markedly retarded and the system preferentially affords (POPy)_4_Cu_4_I_4_-β crystals. Single crystal X-ray (SC-XRD) diffraction analysis at room temperature (RT) reveals that (POPy)_4_Cu_2_I_2_, (POPy)_4_Cu_4_I_4_-α and (POPy)_4_Cu_4_I_4_-β crystallize in the monoclinic *P*2_1_/*n*, tetragonal *P*4̄, and monoclinic *C*2/*c* space groups, respectively (Fig. S1). The crystal structures of the three compounds are shown in Fig. 1a–c, and the detailed crystallographic data are collected in Table S1. As shown in Fig. 1a, the organic ligands combine with Cu–I centers to form a binuclear butterfly-shaped [Cu_2_I_2_] cluster, in which each Cu atom is coordinated simultaneously with the nitrogen atoms of two POPy ligands to form a robust tetrahedral coordination geometric structure. Fig. 1b and c reveal that (POPy)_4_Cu_4_I_4_-α/β are a pair of isomers with the same chemical formula, in which each copper atom coordinates with a nitrogen atom to present a tetrahedral NCuI_3_ geometric environment, eventually forming a classical cubic structure with four copper atoms and four iodine atoms alternately occupying the corner of a twisted cube. According to the connectivity modes of organic and inorganic modules ranging from discrete clusters to extended structures of higher dimension, the three compounds are separated into relatively independent units by organic ligands, featuring zero-dimensional structures. Table S2 lists the Cu–Cu bond lengths in the three compounds, and it can be seen that the Cu–Cu bond distances in (POPy)_4_Cu_2_I_2_ are shorter (2.76 Å) than twice the van der Waals radius of Cu (2.80 Å), indicating the existence of Cu–Cu bonding interactions.^31^ For (POPy)_4_Cu_4_I_4_-α/β, the average Cu–Cu bond lengths are 2.67 and 2.70 Å, respectively, disclosing strong cuprophilic bonding interactions in [Cu_4_I_4_] cubanes, which may promote the formation of ^3^CC excited states. The purity of the four compounds was verified by powder X-ray diffraction (PXRD). As shown in Fig. S2, the PXRD patterns of the three compounds are in excellent agreement with the simulation profiles of single crystal data. The state of the Cu ion was measured and analyzed by X-ray photoelectron spectroscopy (XPS). As shown in Fig. S3, the Cu 2p orbital curve of XPS produces two peaks at 931.8 and 951.8 eV, confirming the +1 oxidation state of Cu. Thermal gravimetric analysis (TGA) indicates that (POPy)_4_Cu_4_I_4_-α/β begin to lose weight when heated to 170 °C in a nitrogen atmosphere, while (POPy)_4_Cu_2_I_2_ shows a lower stability and remains stable until approximately 130 °C (Fig. S4). The superior thermal stability of these Cu_4_I_4_ clusters may be related to their cubic skeleton, as this structure is geometrically rigid.

To elucidate the causes of the formation of the different phases, the crystal data of (POPy)_4_Cu_4_I_4_-α/β are analyzed in detail. It is worth noting that the configurations of ligand molecules are significantly different in (POPy)_4_Cu_4_I_4_-α/β, where the benzene rings of POPy show varying degrees of deflection. In ligand POPy, the connection between the benzene ring and the pyridine ring is achieved by oxygen atoms, which can be regarded as a flexible chain and allows a certain degree of torsion freedom between the two rings. The dihedral angles between the benzene and pyridine ring planes in each ligand POPy of the (POPy)_4_Cu_4_I_4_-α/β are collected to describe the degrees of deflection (Table S3). Fig. S5 displays the smallest asymmetric units of the obtained Cu_4_I_4_ clusters. It can be seen that (POPy)_4_Cu_4_I_4_-α exhibits a high structural symmetry, where the conformation of ligand POPy is single, with a dihedral angle of 65.67° (Fig. 1b). Whereas for (POPy)_4_Cu_4_I_4_-β, there are two types of POPy ligands with different conformations, which deliver larger dihedral angles than the counterparts in the α-phase, that is, 70.30° and 87.15°, respectively (Fig. 1c). Obviously, the differentiated dihedral angles lead to a lower symmetry in (POPy)_4_Cu_4_I_4_-β. Here, it can be speculated that such distortions of organic ligands result in the formation of different α/β-phases. In addition, the deflection of the benzene rings of POPy has significant effects on the electronic structure of the whole cluster (^3^M/XLCT structure for Cu_4_I_4_-α and ^3^CC structure for Cu_4_I_4_-β), as it participates in the charge transition and affects the luminescent properties, which has been confirmed and discussed in the electronic structures part below. It is worth noting that, in contrast to (POPy)_4_Cu_4_I_4_-α, the Cu_4_I_4_ skeleton of (POPy)_4_Cu_4_I_4_-β displays a greater degree of distortion for balancing the larger dihedral angle deflections on the ligand molecules. The polyhedral distortion index (*D*_tet_ and *σ*_tet_^2^) could be utilized to quantify the distortive degree of NCuI_3_ tetrahedra using the following formulae (eqn (1) and (2)):^32^1
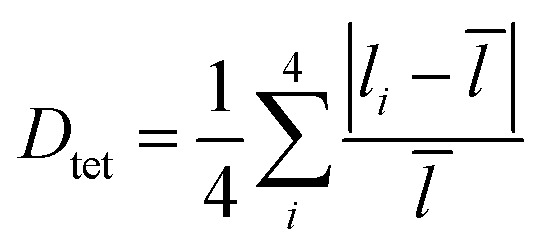
2
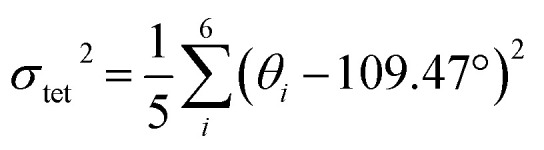
where *l̄*, *l*_*i*_ and *σ*_*i*_ represent the average Cu–I/N bond length, the individual Cu–I/N bond length and the individual I–Cu–I/N bond angle of the tetrahedral unit, respectively. The selected bond lengths and angles are listed in Tables S4 and S5, and the results of distortion index are collected in Table S6. One can see that the polyhedral distortion index of (POPy)_4_Cu_4_I_4_-α (*D*_tet_ = 9.898 × 10^−2^, *σ*_tet_^2^ = 10.023) is obviously smaller than that of (POPy)_4_Cu_4_I_4_-β (*D*_tet_ = 1.019 × 10^−1^, *σ*_tet_^2^ = 27.295). Here, it is reasonable to infer that the ^3^M/XLCT structure is characterized with high symmetry, while ^3^CC structure features marked reduction in the symmetry and dramatic distortion. Very recently, Ihee *et al.* utilized time-resolved X-ray liquidography to investigate the excited-state structural dynamics of Cu_4_I_4_(py)_4_, and achieved conclusions similar to ours.^33^ Our work obtained isomeric Cu_4_I_4_ clusters with ^3^M/XLCT and ^3^CC characteristics respectively under ambient conditions, providing an excellent template for understanding the excited-state structural dynamics of these clusters. In addition, such ligand design gives inspiration for regulating intramolecular charge transfer through conformation engineering.

### Photophysical properties

**Fig. 2:**
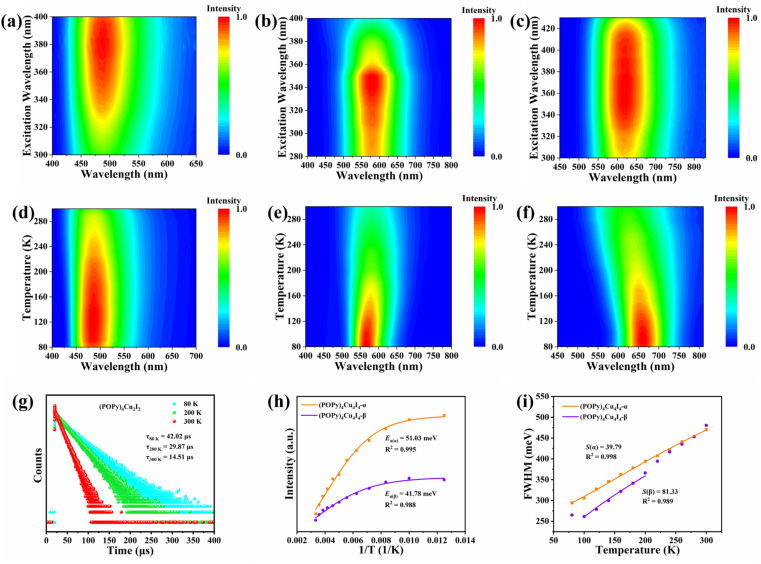
Excitation-dependent emission spectra of (a) (POPy)_4_Cu_2_I_2_, (b) (POPy)_4_Cu_4_I_4_-α and (c) (POPy)_4_Cu_4_I_4_-β. Temperature-dependent photoluminescence spectra of (d) (POPy)_4_Cu_2_I_2_, (e) (POPy)_4_Cu_4_I_4_-α and (f) (POPy)_4_Cu_4_I_4_-β. (g) PL decay lifetime curves of (POPy)_4_Cu_2_I_2_ at 80, 200 and 300 K. (h) Fitting results of the intensity as a function of temperature for (POPy)_4_Cu_4_I_4_-α and (POPy)_4_Cu_4_I_4_-β. (i) FWHM as a function of temperature for (POPy)_4_Cu_4_I_4_-α and (POPy)_4_Cu_4_I_4_-β.

Benefiting from the diverse configurations of the organic and inorganic components, (POPy)_4_Cu_2_I_2_, (POPy)_4_Cu_4_I_4_-α, and (POPy)_4_Cu_4_I_4_-β exhibit rich luminescent properties. As shown in Fig. 1e, (POPy)_4_Cu_2_I_2_, (POPy)_4_Cu_4_I_4_-α, and (POPy)_4_Cu_4_I_4_-β emit blue, yellow and red light under 365 ultraviolet (UV) lamp irradiation, respectively, almost covering the entire visible range. The optical bandgaps of the three compounds are determined by diffuse reflectance spectroscopy (Fig. S6), where the direct bandgaps are estimated to be 2.85, 3.19 and 2.89 eV for (POPy)_4_Cu_2_I_2_, (POPy)_4_Cu_4_I_4_-α, and (POPy)_4_Cu_4_I_4_-β, respectively. As shown in Fig. 1d, (POPy)_4_Cu_2_I_2_ exhibits a wide range of absorption from 300 to 600 nm, which means a strong self-absorption and is not beneficial for emission. The experimental absorption edges of both (POPy)_4_Cu_4_I_4_-α/β are close to their excitation peak maxima (Fig. 1d and e). The photoluminescence and PL excitation (PLE) spectra of the three compounds at RT are characterized by steady-state spectroscopy. As shown in Fig. 1e, (POPy)_4_Cu_2_I_2_ exhibits a broadband emission centered at 488 nm under an excitation wavelength of 372 nm, with a full width at half-maximum (FWHM) of 110 nm and a Stokes shift of 116 nm. Under 350 nm excitation, the PL spectrum of (POPy)_4_Cu_4_I_4_-α shows a strong broadband emission peak at 584 nm with a FWHM of 131 nm and a large Stokes shift of 234 nm. (POPy)_4_Cu_4_I_4_-β produces a broadband emission centered at 620 nm with a FWHM of 149 nm and a Stokes shift of 248 nm under 372 nm excitation. The large Stokes shifts of (POPy)_4_Cu_4_I_4_-α/β indicate negligible self-absorption, which are favorable for efficient luminescence. The photoluminescence quantum yields (PLQYs) of (POPy)_4_Cu_2_I_2_, (POPy)_4_Cu_4_I_4_-α, and (POPy)_4_Cu_4_I_4_-β are measured at RT and determined to be 48.14%, 99.85% and 39.95%, respectively (Fig. S7). Due to the superior PLQY, (POPy)_4_Cu_4_I_4_-α is a preference for lighting applications. According to the PL spectra of the three compounds, the CIE coordinates are calculated to be (0.22, 0.34) for (POPy)_4_Cu_2_I_2_, (0.46, 0.48) for (POPy)_4_Cu_4_I_4_-α and (0.55, 0.44) for (POPy)_4_Cu_4_I_4_-β (Fig. S8). The time-resolved PL decay curves of the three compounds are collected at RT to further investigate their photophysical properties. By single exponential fitting, the lifetimes of (POPy)_4_Cu_2_I_2_, (POPy)_4_Cu_4_I_4_-α, and (POPy)_4_Cu_4_I_4_-β are determined to be 14.13 μs, 10.86 μs and 16.53 μs, respectively (Fig. 1f). These microsecond long lifetimes indicate that the radiative relaxation originates from the recombination of triplet states.^34^ The PL spectra at different excitation wavelengths are measured at RT, as shown in Fig. 2a–c, and there is only one emission center in each three-dimensional continuous PL mapping, which indicates that their radiative paths are single.

To further elucidate the photophysical processes, temperature-dependent PL spectra have been measured for the three compounds. It can be observed in Fig. 2d and S9 that the emission intensity of (POPy)_4_Cu_2_I_2_ is gradually enhanced with the temperature increasing from 80 to 130 K. In the temperature range of 130–300 K, the emission of (POPy)_4_Cu_2_I_2_ exhibits a thermal quenching behavior. The emission enhancement behavior of (POPy)_4_Cu_2_I_2_ in the temperature range from 80 K to 130 K is called thermal activation. This demonstrates the existence of a certain energy barrier Δ*E*, which can be calculated according to the following formula (eqn (3)):^35^

3 Δ*E* = *k*_B_*T*

where *k*_B_ is the Boltzmann constant, *T* = 130 K, and Δ*E* is calculated to be 11.2 meV. As shown in Fig. 2g, the lifetimes for (POPy)_4_Cu_2_I_2_ are gradually increasing with decreasing temperatures and are fitted by single exponential, delivering triplet phosphorescent characteristics. Subsequently, it is found that the PL spectra of (POPy)_4_Cu_4_I_4_-α/β have only one emission peak in the temperature range of 80–300 K (Fig. 2e and f), indicating the single emissive mechanism. In addition, the maximum emission wavelength of (POPy)_4_Cu_4_I_4_-α is gradually blue-shifted with the decrease in test temperature, while that of (POPy)_4_Cu_4_I_4_-β is red-shifted at low temperature. The phenomenon that the emission peak position is temperature-dependent has been observed in many Cu(i)-based materials, and is usually attributed to the influence of lattice thermal expansion.^36^ The intensity of the emission peak of (POPy)_4_Cu_4_I_4_-α/β decreases gradually with the broadening of bandwidths during the temperature increase from 80 to 300 K, which is attributed to the nonradiative decay.^37^

To deeply examine the PL distinctions in (POPy)_4_Cu_4_I_4_-α/β, the Arrhenius formula is used to calculate the exciton activation energy (*E*_a_), deduced as eqn (4):^38^4
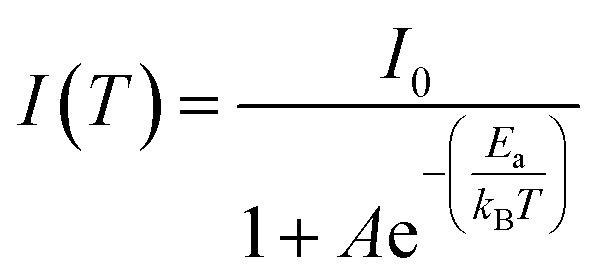
where *I*(*T*) and *I*_0_ represent the PL intensity at 0 K and measured temperature, respectively, and *k*_B_ denotes the Boltzmann constant. As shown in Fig. 2h, the *E*_a_ of (POPy)_4_Cu_4_I_4_-β is determined to be 41.78 meV, a bit smaller than that of (POPy)_4_Cu_4_I_4_-α (51.03 meV). The large values of *E*_a_ are conducive for the capture of excitons, thereby promoting radiation decay. Considering broadband emission of (POPy)_4_Cu_4_I_4_-α/β, the relationships between FWHM and temperature are fitted by using the following formula (eqn (5)):^39^5
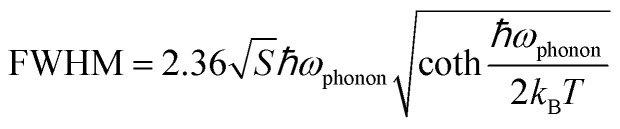
where *ħω*_phonon_ means the phonon energy and *S* denotes the Huang–Rhys factor. As shown in Fig. 2i, the FWHM of both (POPy)_4_Cu_4_I_4_-α/β becomes broad with increasing temperature, indicating an enhancement of electron–phonon coupling. The *S* value can be estimated to be 39.79 for (POPy)_4_Cu_4_I_4_-α and 81.33 for (POPy)_4_Cu_4_I_4_-β. The larger *S* value of (POPy)_4_Cu_4_I_4_-β means a stronger electron–phonon coupling, which corresponds to its larger degree of structural distortion to promote Jahn–Teller excited state distortion. The ^3^M/XLCT structure of (POPy)_4_Cu_4_I_4_-α presents a spatially separated electron–hole distribution, which reduces exciton–phonon coupling and mitigates energy loss caused by Jahn–Teller distortion, eventually culminating in a near-unity PLQY.

### Electronic structure

**Fig. 3:**
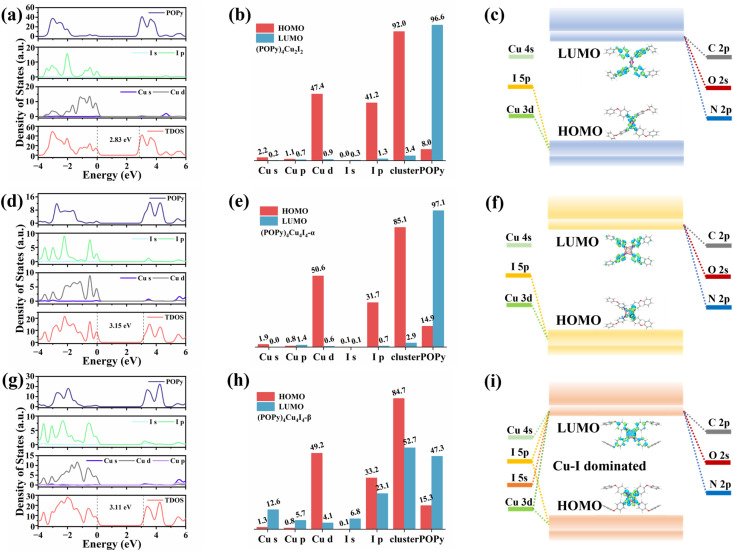
Density of states, orbital composition analysis (the numerical values denote the percentage contributions of different Cu/I/POPy shells to the HOMO and LUMO, %) and frontier energy band diagrams for (a–c) (POPy)_4_Cu_2_I_2_, (d–f) (POPy)_4_Cu_4_I_4_-α and (g–i) (POPy)_4_Cu_4_I_4_-β.

We further performed DFT calculations to gain insights into the mechanism of the optical behaviors of the three compounds. As shown in Fig. S10, flat band edges can be observed on both sides of the valence and conduction bands of the three compounds, indicating that there is almost no overlap of wave functions and electronic coupling between adjacent clusters.^40^ The bandgaps calculated by DFT are 2.83, 3.15 and 3.11 eV for (POPy)_4_Cu_2_I_2_, (POPy)_4_Cu_4_I_4_-α, and (POPy)_4_Cu_4_I_4_-β, respectively, which are in reasonable agreement with experimentally measured optical bandgaps, considering the challenge of accurate prediction of bandgaps of organic–inorganic hybrid semiconductors by standard DFT methods and the difference of definition between optical bandgaps and HOMO–LUMO (the highest occupied molecular orbital – the lowest unoccupied molecular orbital) gaps.^41^ The charge density distributions of the three compounds are shown in Fig. S11. It can be clearly seen that the inorganic and organic parts of the three compounds are involved in the composition of the HOMO and LUMO. Fig. 3a–c present the density of states (DOS), orbital composition analysis and electronic charge densities of (POPy)_4_Cu_2_I_2_. It can be seen that the HOMO is localized in the [Cu_2_I_2_] core, primarily contributed by the Cu 3d and I 5p orbitals, whereas the LUMO is localized in the organic ligands. In (POPy)_4_Cu_4_I_4_-α, the HOMO primarily consists of Cu 3d and I 5p orbitals, while the orbitals of organic components dominate the LUMO (Fig. 3d and e). Similar to (POPy)_4_Cu_2_I_2_, the HOMO and LUMO of (POPy)_4_Cu_4_I_4_-α are localized in the [Cu_4_I_4_] core and ligands (Fig. 3f), respectively. Therefore, the emissions from (POPy)_4_Cu_2_I_2_ and (POPy)_4_Cu_4_I_4_-α can be assigned to ^3^M/XLCT, which are consistent with those of previously reported Cu-based clusters with [Cu_2_I_2_] and [Cu_4_I_4_] cores.^42,43^ Although (POPy)_4_Cu_4_I_4_-β shares the same chemical formula and the [Cu_4_I_4_] core with (POPy)_4_Cu_4_I_4_-α, their electronic structures are significantly different. It can be seen in Fig. 3g and h that the Cu-3d electron and I-5p electron play a crucial role in the HOMO, while the 4sp/3d shell of Cu and 5sp states of I atoms dominated in the LUMO, indicating the charge transitions of dominant d → s, p cluster-centered (CC). Such metal-to-iodide states can induce structural distortion in the Cu_4_I_4_ core, which negatively impacts the luminescence of clusters. As shown in Fig. 3i, the HOMO and LUMO of (POPy)_4_Cu_4_I_4_-β are partially overlapping, and mainly concentrate on the inorganic [Cu_4_I_4_] core, further suggesting the existence of the CC transition. Here, it is clear that the luminescence of (POPy)_4_Cu_4_I_4_-β is attributed to ^3^CC emission. According to a previous report, ^3^CC emission is typically characterized by a large Stokes shift and low luminous efficiency, which is consistent with the characteristics of (POPy)_4_Cu_4_I_4_-β.^44^ The Jahn–Teller distortion of the ^3^CC structure may increase the overlap of the potential energy surfaces between the excited state and the ground state, ultimately resulting in non-radiative relaxation. Meanwhile, because the charge in the ^3^CC structure is concentrated in the Cu_4_I_4_ core, the radiative recombination channel of the ligand cannot be fully utilized, culminating in a low probability of radiative transition. As a result, the superior luminescent properties of (POPy)_4_Cu_4_I_4_-α under both UV and X-ray irradiations compared to (POPy)_4_Cu_4_I_4_-β can be well understood. Moreover, it is found in the compositional orbital diagram of (POPy)_4_Cu_4_I_4_-β that the ligand also contributes a lot to the LUMO, which means an existence of competition between ^3^CC and ^3^M/XLCT states. Considering that the geometric changes between isomeric (POPy)_4_Cu_4_I_4_-α/β happen in the outer protecting ligands, the distortional degree of organic ligands is associated with their electronic structures, as the large dihedral angle deflection of POPy induces a reduction in the symmetry and a significant distortion in the Cu_4_I_4_ core. Typically, a compact Cu_4_I_4_ unit would restrain further deformation and thereby hamper the formation of the ^3^CC state.^44^ Compared with (POPy)_4_Cu_4_I_4_-α, (POPy)_4_Cu_4_I_4_-β exhibits larger structural distortion and a longer average Cu–Cu distance, yielding a less compact framework. This reduced rigidity facilitates excited-state contraction and deformation, tilting the balance between ^3^M/XLCT and ^3^CC toward the latter. On the basis of the results of spectral characterization and theoretical calculations, the frontier energy band diagrams describing the photophysical processes of (POPy)_4_Cu_2_I_2_, (POPy)_4_Cu_4_I_4_-α and (POPy)_4_Cu_4_I_4_-β are illustrated in Fig. 3c, f and i.

To further elucidate the structure–property relationship of the Cu_4_I_4_ cluster, we characterized the excited-state structures of (POPy)_4_Cu_4_I_4_-α and (POPy)_4_Cu_4_I_4_-β by time-dependent DFT (TD-DFT). We examined the Δ*Q* (the changes between the ground- and excited-state equilibrium position) to reflect the distortion degree in the lowest triplet (T_1_) excited states relative to the ground state (S_0_) within a periodic unit.^45^ As depicted in Fig. S12a, (POPy)_4_Cu_4_I_4_-α exhibits a small structural change from S_0_ to T_1_. Whereas for (POPy)_4_Cu_4_I_4_-β, the Δ*Q* is much higher than that of (POPy)_4_Cu_4_I_4_-α (Fig. S12b), indicating a larger degree of excited-state structural distortion. This might be due to the fact that the highly symmetrical cubic Cu_4_I_4_ configuration of (POPy)_4_Cu_4_I_4_-α is more resistant to the excited-state deformation, attributed to its robust structural rigidity. The lower excited-state distortion significantly suppresses non-radiative recombination, enabling a pronounced PLQY of up to 99% in (POPy)_4_Cu_4_I_4_-α. The intense Jahn–Teller distortion in (POPy)_4_Cu_4_I_4_-β leads to a decrease in emission energy, which is consistent with the trend of the Huang–Rhys factor.

### X-ray scintillation properties

**Fig. 4:**
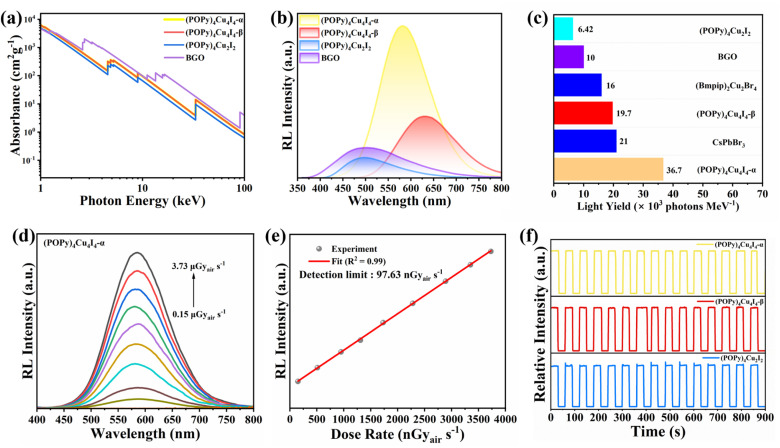
(a) Absorption coefficients of the obtained complexes and BGO as a function of photon energy. (b) RL spectra of the obtained complexes and BGO under the same conditions. (c) A comparison of light yield between the obtained complexes and conventional scintillators. (d) RL spectra of (POPy)_4_Cu_2_I_2_ under X-ray radiation at different dose rates. (e) Linear fitting of RL response intensity *vs.* dose rate. (f) The irradiation stability of the obtained compounds under cyclic X-ray illumination.

The excellent photophysical properties of the three compounds, including relatively short lifetime and broadband emission with high PLQY, inspire us to explore their potential application as X-ray scintillators. According to the XCOM web database, we calculated the absorption coefficients of the three compounds and the commercial Bi_4_Ge_3_O_12_ (BGO) scintillator at different photon energies.^46^ As shown in Fig. 4a, the X-ray absorption coefficients of the three compounds are slightly lower than that of BGO, indicating that they have strong X-ray absorption capacity in the extensive energy range. Similar to the PL properties under UV light excitation, the radioluminescence spectra of (POPy)_4_Cu_4_I_4_-α exhibit the same yellow emission, indicating the same luminescence mechanism (Fig. S13b). Under X-ray irradiation, (POPy)_4_Cu_2_I_2_ exhibits an emission peak at 496 nm with a FWHM of 128 nm (Fig. S13a), and a redshifted and wider spectrum compared with the PL spectrum shown in Fig. 1e. A similar redshift phenomenon also exists in (POPy)_4_Cu_4_I_4_-β (Fig. S13c). The red shifts observed in the RL spectrum may be attributed to the radiative recombination of shallow defects near the LUMO.^47^ In order to evaluate the ability of the three compounds to convert X-rays into visible light, we compared the integrated area of their RL emission spectra with BGO as a reference (Fig. 4b). As illustrated in Fig. 4c, the light yields of (POPy)_4_Cu_2_I_2_, (POPy)_4_Cu_4_I_4_-α, and (POPy)_4_Cu_4_I_4_-β were calculated to be 6420, 36 700 and 19 700 photons per MeV, respectively. The light yield of (POPy)_4_Cu_4_I_4_-α is much higher than that of the commercial scintillator BGO (10 000 photons per MeV), and also higher than those of the reported metal halide scintillators, including (Bmpip)_2_Cu_2_Br_4_ (16 000 photons per MeV) and CsPbBr_3_ (21 000 photons per MeV).^48^ Such high light yields benefit from high PLQY, negligible self-absorption, and large X-ray absorption cross section of the heavy [Cu_4_I_4_] core. In contrast, although (POPy)_4_Cu_2_I_2_ possesses ^3^M/XLCT characteristics, its radioluminescence is the most inferior in these clusters, attributed to lower X-ray absorption capacity and strong self-absorption. As illustrated in Fig. 4d, the RL intensity of (POPy)_4_Cu_4_I_4_-α linearly increases with the X-ray dose from 0.15 to 3.73 μGy_air_ s^−1^, indicating the high sensitivity at low doses. As shown in Fig. 4e and S14, the detection limits are calculated to be 0.83 μGy_air_ s^−1^ for (POPy)_4_Cu_2_I_2_, 97.63 nGy_air_ s^−1^ for (POPy)_4_Cu_4_I_4_-α and 132.85 nGy_air_ s^−1^ for (POPy)_4_Cu_4_I_4_-β, which are lower than the value of 5.5 μGy_air_ s^−1^ required for X-ray medical diagnosis.^49^ Subsequently, the irradiation resistance of the three compounds was investigated by exposing them under switched on-off irradiation of high-dose X-rays (Fig. 4f). The results demonstrate that the RL intensity remains unchanged within 15 irradiation on/off cycles for 900 s, indicating excellent X-ray stability. To sum up, considering the high light yield, low detection limit and long-term irradiation stability, (POPy)_4_Cu_4_I_4_-α is considered as an ideal scintillator for X-ray imaging. Building upon these PL and RL properties, it can be speculated that under X-ray excitation, the high-energy electrons generated through the photoelectric effect and Compton scattering relax into secondary electrons. These secondary electrons are transferred to the excited-state energy levels in the materials and achieve efficient ^3^M/XLCT and ^3^CC emissions (Fig. S15).

### X-ray imaging applications

**Fig. 5:**
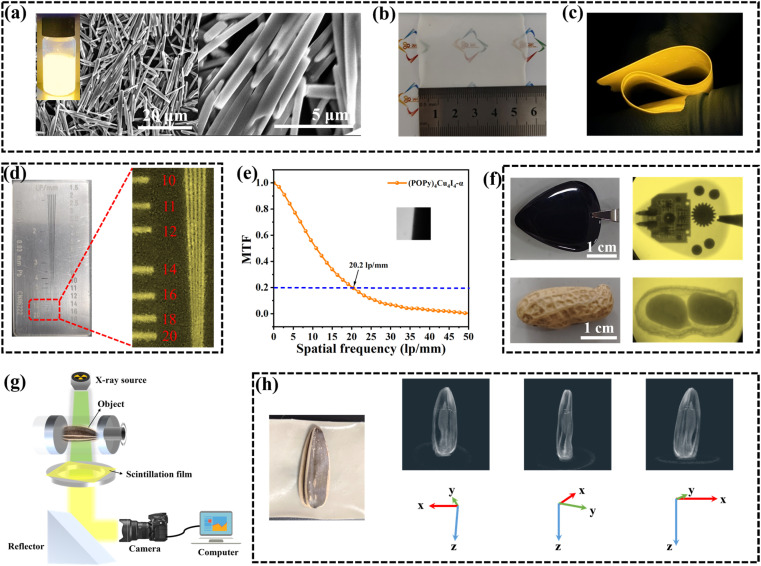
(a) SEM images of (POPy)_4_Cu_4_I_4_-α microcrystals (the inset shows the colloidal cluster microcrystals under UV excitation). (b) Photograph of the assembled flexible film of (POPy)_4_Cu_4_I_4_-α under sunlight. (c) Photograph of bent flexible films under 365 nm UV light. (d) X-ray images of the test-pattern plate. (e) Modulation transfer function (MTF) curve of the flexible film based on the slanted-edge method (inset). (f) Photograph of the target objects and corresponding X-ray images (X-ray dose rate: 179.6 μGy_air_ s^−1^). (g) Schematic diagram of the customized 3D X-ray imaging system. (h) X-ray images and 3D reconstruction of a sunflower seed from multiple perspectives.

Encouraged by the excellent scintillation performance of (POPy)_4_Cu_4_I_4_-α, we fabricated a flexible film as a scintillation screen. The traditional method of fabricating a scintillation screen is to grind the bulk crystals and mix them with the polymer matrix.^50,51^ However, the powders obtained by grinding exhibit the problems of large grain size and non-uniformness in film preparation. To address this issue, we obtained rod-like microcrystals by regulating the crystal growth rate of (POPy)_4_Cu_4_I_4_-α through the surfactant poly(vinylpyrrolidone) (PVP). As shown in Fig. 5a, the well-dispersed microcrystals in methanol exhibit the same yellow luminescence as the bulk crystal. By combining microcrystal inks with a polyvinyl alcohol (PVA) polymer matrix, we fabricated a (POPy)_4_Cu_4_I_4_-α@PVA flexible film with a size of about 5 × 5 cm (Fig. 5b and c). Cross-sectional scanning electron microscopy (SEM) is employed to further characterize the morphology and distribution of microcrystals in the polymer matrix (Fig. S16). It can be clearly seen that the thickness of the film is approximately 80 μm and the microcrystals are uniformly distributed. As shown in Fig. S17, the PXRD pattern of the (POPy)_4_Cu_4_I_4_-α@PVA film is consistent with the simulation result of the (POPy)_4_Cu_4_I_4_-α single crystal, demonstrating the same structure after combination. Furthermore, the flexible film retains a sufficient PLQY of 81.62%, illustrating the excellent luminescence performance (Fig. S18).

Finally, we evaluate the X-ray imaging capability of the (POPy)_4_Cu_4_I_4_-α@PVA film by using a self-built digital radiography system.^52^ A standard X-ray resolution test-pattern plate is used to assess the spatial resolution of the (POPy)_4_Cu_4_I_4_-α@PVA film. As shown in Fig. 5d, a high resolution of 18–20 lp mm^−1^ can be observed. Meanwhile, we employed the slanted-edge method to determine the modulation transfer function (MTF) of the (POPy)_4_Cu_4_I_4_-α@PVA film. The spatial resolution of the (POPy)_4_Cu_4_I_4_-α@PVA film is measured to be 20.2 lp mm^−1^ when MTF equals 0.2, which is close to the above result (Fig. 5e). Benefiting from high resolution, the (POPy)_4_Cu_4_I_4_-α@PVA film can clearly image the target objects. As shown in Fig. 5f, the internal structure of electronic equipment can be clearly distinguished due to the large difference in the absorption capacity of X-rays between plastic shell and electronic components. In addition, the internal structure of peanuts can also be clearly observed, indicating the potential application of bioimaging.

In further experiments, we explored the possible X-ray image fusion as well as 3D image reconstruction. As we know, fusing several X-ray images can obtain the most relevant information from images of different perspectives into a unified 3D model, so as to produce more valuable information than traditional 2D images and meet the requirement of tomography in medical diagnosis. Fig. 5g depicts the schematic diagram of the customized 3D X-ray imaging system, where the object is placed on a rotating plate exposed to the X-ray source and the 2D X-ray images of the target are captured at multiple angles. To realize the 3D imaging, a sunflower seed as a sample was fixed on a rotating plate, and X-ray images were captured at intervals of 5° over a range of 180° using the (POPy)_4_Cu_4_I_4_-α@PVA film. Then, the 3D reconstruction algorithm was employed to process these 2D photos to generate high-quality cross-sectional slices. Finally, a demonstration of 3D reconstruction models was successfully realized by restoring these slices using the AVIZO software (Video 1, SI), disclosing the superb X-ray tomography imaging performance. Fig. 5h presents the selected 3D reconstruction models of the sunflower seed. It can be seen that both the internal structure and the outline of the sunflower seed shell are clearly visible at different angles of rotation around the *Z* axis. These results indicate the great potential of (POPy)_4_Cu_4_I_4_-α in the field of advanced X-ray imaging and detection.

## Conclusion

In summary, we report here a series of new copper(i) coordination compounds, which feature a [Cu_2_I_2_] or a pair of isomeric [Cu_4_I_4_] inorganic modules without changing the organic components. The emission colors of these compounds range from blue and yellow to red, with the highest PLQY of 99.85%. Crystal structure analysis reveals that the conformational changes caused by the flexible O atom linker on ligands is the reason for this configuration-dependent photoluminescence. Spectroscopies and DFT calculations are employed to comprehensively investigate the excited state properties of isomeric (POPy)_4_Cu_4_I_4_-α/β. It has been evidenced that the conformation significantly affects the electronic structures, leading to ^3^M/XLCT transitions for (POPy)_4_Cu_4_I_4_-α and ^3^CC transitions for (POPy)_4_Cu_4_I_4_-β. Moreover, a high-quality X-ray scintillation film is fabricated using (POPy)_4_Cu_4_I_4_-α microcrystals, which exhibits a high spatial resolution of 20.2 lp mm^−1^. Benefiting from the fascinating optical properties of (POPy)_4_Cu_4_I_4_-α, we successfully realize the 3D X-ray imaging by reconstructing the multi-angle images of a sunflower seed as a sample. These results demonstrate that (POPy)_4_Cu_4_I_4_-α has great potential in practical X-ray tomography. Our study introduces a novel approach for designing highly efficient luminophores based on isomeric copper(i) iodide coordination clusters, and also presents a new template for advanced X-ray imaging applications.

## Author contributions

Y. Zhu, S. Liu and Q. Zhao conceived the idea. Y. Zhu prepared the samples, conducted primary testing, and drafted the initial manuscript. Y. Deng helped with the experimental design and contributed to manuscript refinement. N. Ding performed the theoretical calculations. Q. Li assisted with the optical measurements and XRD tests. Y. Wang provided guidance on SEM testing and scintillation film fabrication. M. Wang helped explain the luminescence mechanism. K. Y. Zhang and S. Liu provided valuable suggestions for the experimental data and manuscript revisions. Q. Zhao and S. Liu guided and supervised the experimental design and data analysis and played a crucial role in refining the manuscript.

## Conflicts of interest

There are no conflicts to declare.

## Supplementary Material

SC-016-D5SC06329A-s001

SC-016-D5SC06329A-s002

SC-016-D5SC06329A-s003

## Data Availability

CCDC 2391040–2391042 contain the supplementary crystallographic data for this paper.^53*a*–*c*^ The authors confirm that the data supporting the findings of this study are available within the article or its supplementary information (SI). Supplementary information: experimental procedures, characterization data (SCXRD, SEM, TGA) and additional figures and tables. See DOI: https://doi.org/10.1039/d5sc06329a.
