# Supplementary material for: Regulating charge transfer of copper(i) coordination compounds via conformation engineering for highly efficient radioluminescence and 3D X-ray imaging
Source: Chem Sci. 2025 Oct 22;16(47):22690–700. doi: 10.1039/d5sc06329a (PMC12571119; doi:10.1039/d5sc06329a)
Supplement: SC-016-D5SC06329A-s001 [file SC-016-D5SC06329A-s001.pdf]

Supplementary Information (SI) for Chemical Science.
This journal is © The Royal Society of Chemistry 2025

Supporting Information

Regulating charge transfer of copper(I) coordination compounds via conformation engineering for highly-efficient radioluminescence and 3D X-ray imaging

Yongkang Zhu,[a] Yongjing Deng,[a] Qianxi Li,[a] Ning Ding,[a] Yulong Wang,[a] Mengzhu Wang,[a] Kenneth Yin Zhang,[a] Shujuan Liu,[a]* and Qiang Zhao[a,b]*

[a] Y. Zhu, Y. Deng, Q. Li, N. Ding, Y. Wang, M. Wang, Prof. K. Y. Zhang, Prof. S. Liu, Prof. Q. Zhao

State Key Laboratory of Flexible Electronics (LoFE), Institute of Advanced Materials (IAM) & Institute of Flexible Electronics (Future Technology)

Nanjing University of Posts & Telecommunications

Nanjing 210023, P. R. China

[b] Prof. Q. Zhao

School of Electronics and Information Technology

Nanjing University of Information Science and Technology

Nanjing 210044, China

E-mail: iamsjliu@njupt.edu.cn (S. Liu), iamqzhao@nuist.edu.cn (Q. Zhao)

Experimental Procedures

Materials

Cuprous iodide (CuI, 99%, Adamas), 4-phenoxypyridine (POPy, 98%, Meryer), potassium iodide (KI, AR, HUSHI), deionized water, methanol (MeOH, AR, HUSHI), ethanol (EtOH, AR, HUSHI), acetonitrile (AR, Energy), polyvinylpyrrolidone (PVP, M.W. 1300000, Aladdin) and Poly(vinyl alcohol) 1799. All chemicals were purchased and used without further purification.

General Experimental Information

Synthesis of $(POPy)_4Cu_2I_2$ crystals. Single crystals of $(POPy)_4Cu_2I_2$ were grown by diffusion method in a 20 ml glass bottle. A saturated KI aqueous solution (2 ml) containing CuI (1 mmol) was placed in bottom layer of glass bottle. Then, 4 ml acetonitrile was slowly and carefully added as the buffer layer. Finally, a mixture of ethanol (2 ml) and 4-phenoxypyredine (2 mmol) was placed on the top layer. After standing for 1 day, the colorless, block crystals were obtained.

Synthesis of $(POPy)_4Cu_4I_4$-α crystals. The synthesis method of $(POPy)_4Cu_4I_4$-α single crystals was similar to $(POPy)_4Cu_2I_2$. The difference was that the content of 4-phenoxypyredine in the top layer solution was 1 mmol. After standing for 1 day, the colorless, needle-like crystals were obtained.

Synthesis of $(POPy)_4Cu_4I_4$-β crystals. Single crystals of $(POPy)_4Cu_4I_4$-β were grown by slow diffusion method in a 20 ml glass bottle. An acetonitrile solution (5 ml) containing CuI (0.5 mmol) was placed in bottom layer of glass bottle; then, a mixture of acetone (3 ml) and 4-phenoxypyridine (0.5 mmol) was slowly added from the top layer. After standing for 1 day, the colorless, needle-like crystals were separate by hand picking under 365 nm ultraviolet lamp.

Fabrication of the $(POPy)_4Cu_4I_4$-α microcrystal ink. CuI (0.2 mmol) was dissolved into acetonitrile (2 mL). Meanwhile, 0.2 g polyvinylpyrrolidone (PVP) was dissolved into the mixed solvent of methanol (20 mL). 0.2 mmol POPy was dropped into PVP solution. After that, the CuI precursor solution was slowly added under vigorous stirring (800 rpm). The white colloid could be formed immediately. After 24 hours of stirring, the microcrystals were collected by centrifugation at 10,000 rpm for 5 minutes and washed twice with methanol.

Fabrication of the X-ray scintillator screen. 1.2 g of PVA was completely dissolved in deionized water (10 mL) to form solution under stirring at 90 ℃ for 5 hours. Then 1 mL of water solution of $(POPy)_4Cu_4I_4$-α

microcrystals was added into the above solution under stirring until the formation of a homogeneous and viscous fluid. The final flexible film was prepared by facilely coating on a glass and being evaporated slowly in the ventilation cabinet at room temperature.

Characterization

The single-crystal X-ray diffraction SCXRD data were obtained on the Bruker Smart Apex CCD diffractometer at room temperature using ω-2θ scanning technique with graphite monochromatic Mo-Kα (λ = 0.71073 Å) as the radiation source. The single crystal structure is solved and refined by ShelXL and Olex2 (the full small square method F^2). The crystallographic data for the structural analysis have been deposited in the Cambridge Crystallographic Data Center with the CCDC reference numbers of 2391040, 2391041 and 2391042. X-ray powder diffraction (PXRD) using the X-ray diffractometer D8 Advance A25 with Cu K_α radiation (λ = 1.5406 Å).

Optical Performance Measurements

The UV–vis absorption spectra were recorded by a SHIMADZU UV-2600 spectrophotometer equipped with an ISR-2600Plus integrating sphere and $BaSO_4$ was used for 100% reflectance reference. Excitation spectra, and steady-state spectrum and time-resolved decay spectra were measured on FLS980 spectrophotometer (Edinburgh instrument) equipped with a 450 W xenon lamp and a 375 nm laser as the excitation source. The PLQY was measured by Edinburgh FLS980 equipped with an integrating sphere. All the measurements about photoluminescence radioluminescence were carried out under an ambient air atmosphere.

Thermogravimetric Analysis (TGA)

The TGA data was obtained through the NETZSCH STA-2500 at rate of 10 °C/min under pure nitrogen atmosphere (25-500 °C).

Scanning Electron Microscopy (SEM)

The morphology and microstructure of samples were characterized by SEM (Hitachi S-4800).

DFT calculations

All DFT calculations were carried out with the CP2K package version-2024.1 using Gaussian Plane Wave (GPW) method implemented in the QUICKSTEP module.[1,2] The Perdew-Burke-Ernzerhof (PBE) exchange-correlation (XC) functional with Grimme-D3 (Becke-Johnson, BJ) dispersion correction method was employed in the calculations of geometry optimization and band structure.[3,4] The Brillouin zone integration was performed with 3 × 2 × 2 Γ-centered Monkhorst-Pack k-point meshes for $(POPy)_4Cu_2I_2$, 2 × 2 × 2 for $(POPy)_4Cu_4I_4$-α and 1

× 2 × 1 for $(POPy)_4Cu_4I_4$-β. The Heyd-Scuseria-Ernzerhof hybrid (HSE06) XC functional with Grimme-D3 (BJ) dispersion correction method was employed in the calculations of density of states (DOS) and charge density distributions for HOMO and LUMO with a Γ-point-only sampling (1 × 1 × 1 k-point mesh) and a 3 × 2 × 2 supercell model for $(POPy)_4Cu_2I_2$, 2 × 2 × 2 for $(POPy)_4Cu_4I_4$-α and 1 × 2 × 1 for $(POPy)_4Cu_4I_4$-β.[5] The bandgap values were derived from DOS calculations. The GTH-PBE pseudopotential and the TZVP-MOLOPT-GTH basis set were adopted for C, H, O and N elements, while for Cu and I, the TZVP-MOLOPT-SR-GTH basis set was used.[6] Plane wave and relative cut-offs were set to 400 and 55 Ry, respectively. Time-dependent DFT (TDDFT) calculations was conducted for the geometry optimization of the lowest triplet state (T_1) with a Γ-point-only sampling. The supercell models used in TDDFT calculations were same as which in DOS calculations. The excited-state structural distortions was calculated by $\Delta Q = \sqrt{\sum_{\kappa,i} M_\kappa (R^e_{\kappa,i} - R^g_{\kappa,i})^2}$, where κ denoted the atom, *i* = (x, y, z), *M* was the atomic mass and *R* were the atomic coordinates with e and g for the T_1 and ground state, respectively. The calculated ΔQ of $(POPy)_4Cu_4I_4$-α and $(POPy)_4Cu_4I_4$-β were 11.83 and 16.32 Å $AMU^{1/2}$, respectively. The visual molecular dynamics (VMD) program was employed for the visualization and analysis of the results.[7] Multiwfn software was employed for the generation of input file of CP2K and the electronic structure analysis.[8]

Determination of X-ray excited relative light yields:

The X-ray relative light yields in this work were measured by using commercial BGO as a reference. The samples (about 100 μm) of the prepared materials and the reference scintillator (BGO) were set at the same position to measure the XEL spectra. The corresponding photon counting results ($PC_{measured}$) were then obtained by integrating the steady-state XEL spectra. Compared with reference BGO, the corresponding relative light yields were calculated according to equation (1) and (2). The equations were as follows:

$$PC_{normalized} = \frac{PC_{measured}}{AE(d)} \quad (1)$$

$$LY_s = LY_{BGO} \frac{PC_{normalized}(S)}{PC_{normalized}(BGO)} \quad (2)$$

where LY_{BGO} is the light yield of BGO (10000 photons MeV^{-1}), $PC_{normalized}$ (S) and $PC_{normalized}$ (BGO) are the photon counts of our scintillators and BGO normalized to respective X-ray attenuation efficiencies.

Calculation of X-ray attenuation efficiency:

The X-ray attenuation efficiency (AE) of the prepared materials and BGO were calculated by equation (3) and (4) combining the output spectra of the X-ray source (Au target with output energy range from 1.15 keV to 69.6 keV).

$$AE(\%) = \left(1 - e^{-c(\varepsilon)\rho d}\right) \times 100\% \tag{3}$$

$$AE(d) = \int_{1.15}^{69.6} \frac{AE(\varepsilon, d) \times R(\varepsilon) d\varepsilon}{(\int_{1.15}^{69.6} R(\varepsilon) d\varepsilon} \tag{4}$$

where AE(d) is the X-ray attenuation efficiency (%) of scintillators for the entire X-ray photons energy range (from 1 keV to 100 MeV) at a certain thickness (100 μm), *c(ε)* is the X-ray absorption coefficients derived from the XCOM database of the National Institute of Standards and Technology, *ρ* is the density of the material and *d* is the thickness, *R(ε)* is the output spectra of the X-ray tube. *R(ε)* and *c(ε)* were calculated after being differentiated by 50,000 copies.

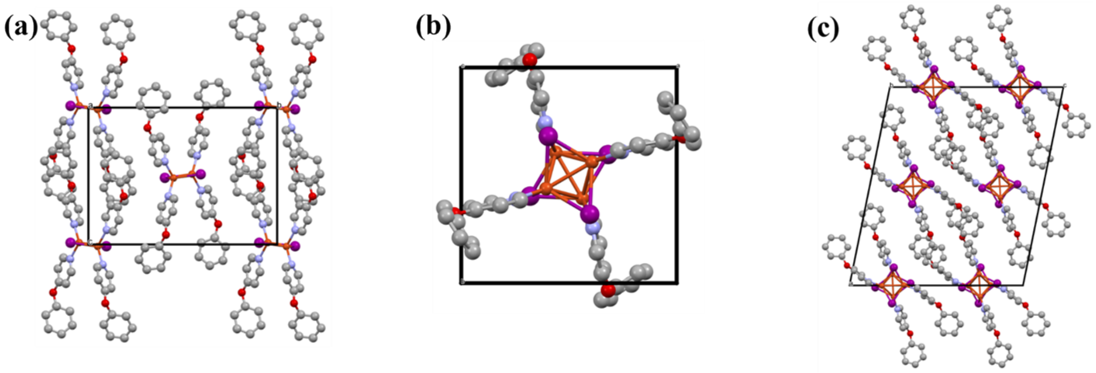

Fig. S1. Packing crystal structures of (a) $(POPy)_4Cu_2I_2$, (b) $(POPy)_4Cu_4I_4$-α and (c) $(POPy)_4Cu_4I_4$-β.

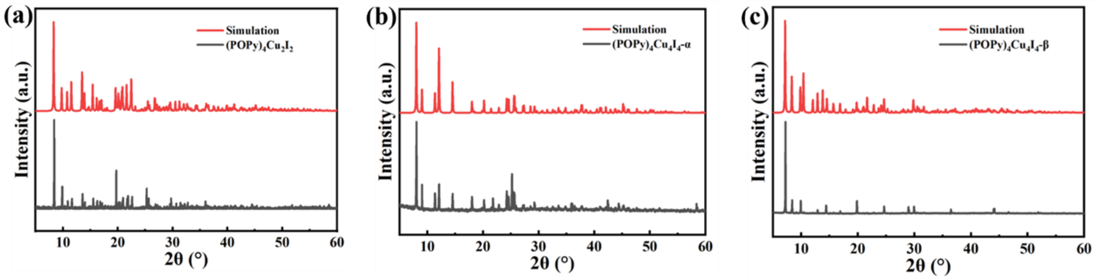

Fig. S2. PXRD patterns and the corresponding simulated profiles from single crystal structures of (a) $(POPy)_4Cu_2I_2$, (b) $(POPy)_4Cu_4I_4$-α and (c) $(POPy)_4Cu_4I_4$-β.

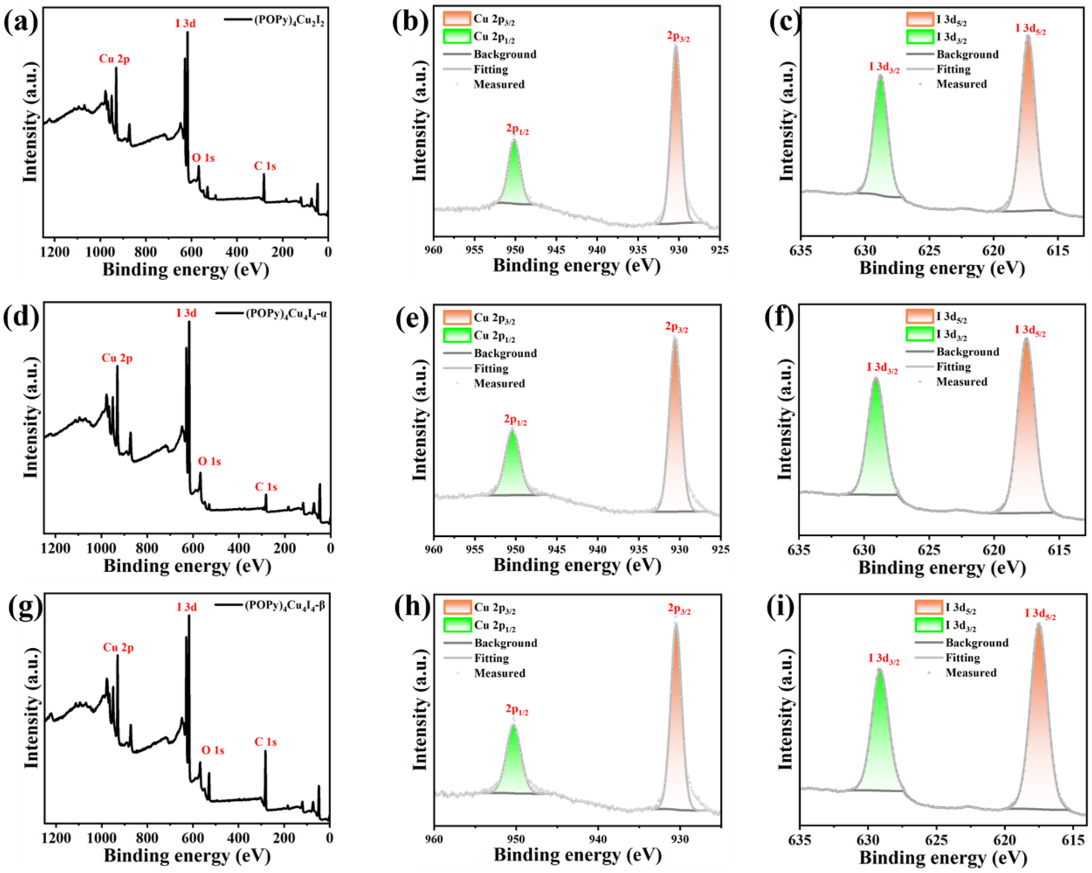

Fig. S3. XPS spectra of (a-c) $(POPy)_4Cu_2I_2$, (d-f) $(POPy)_4Cu_4I_4$-α and (g-i) $(POPy)_4Cu_4I_4$-β.

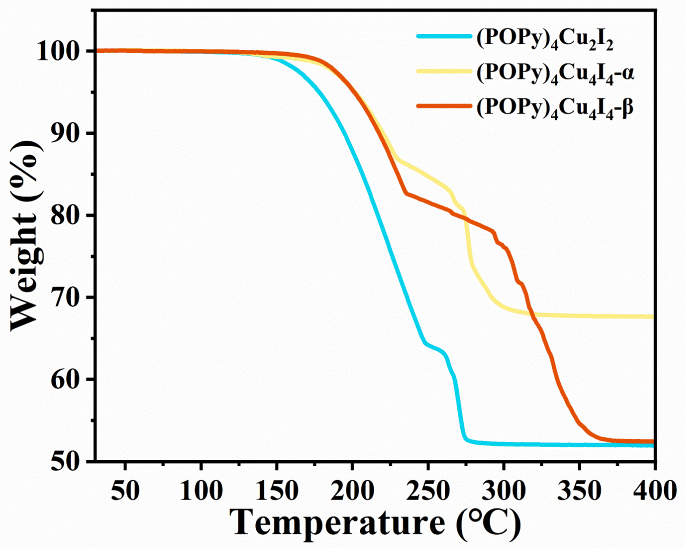

Fig. S4. TGA curves of $(POPy)_4Cu_2I_2$, $(POPy)_4Cu_4I_4$-α and $(POPy)_4Cu_4I_4$-β.

(a) (b)

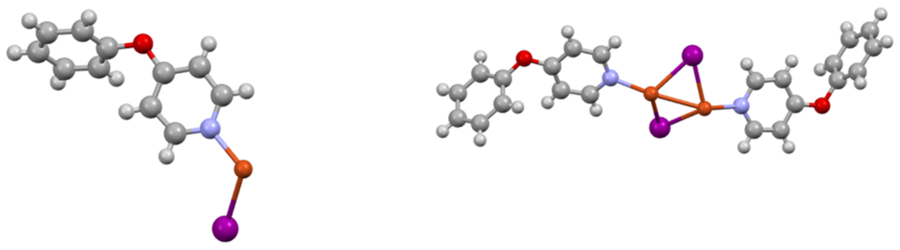

Fig. S5. The smallest asymmetric units of $(POPy)_4Cu_4I_4$-α and $(POPy)_4Cu_4I_4$-β.

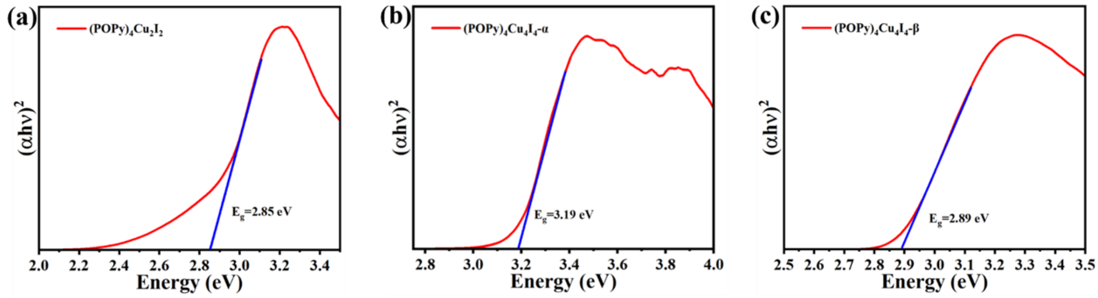

Fig. S6. The Tauc's plot based on the assumption of direct band gap of (a) $(POPy)_4Cu_2I_2$, (b) $(POPy)_4Cu_4I_4$-α and (c) $(POPy)_4Cu_4I_4$-β.

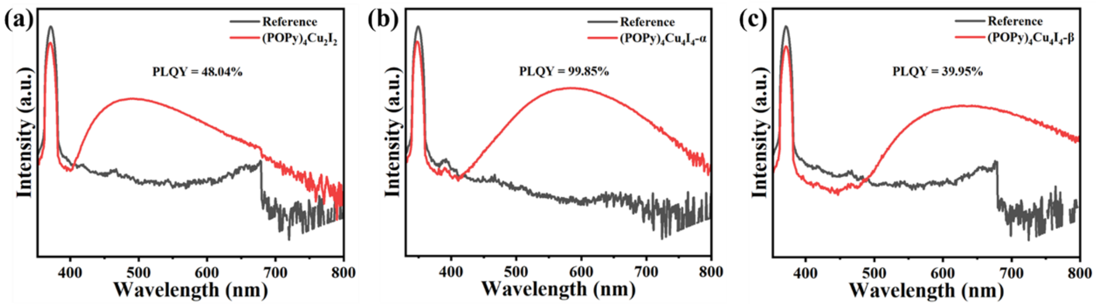

Fig. S7. The PLQYs of (a) $(POPy)_4Cu_2I_2$, (b) $(POPy)_4Cu_4I_4$-α and (c) $(POPy)_4Cu_4I_4$-β.

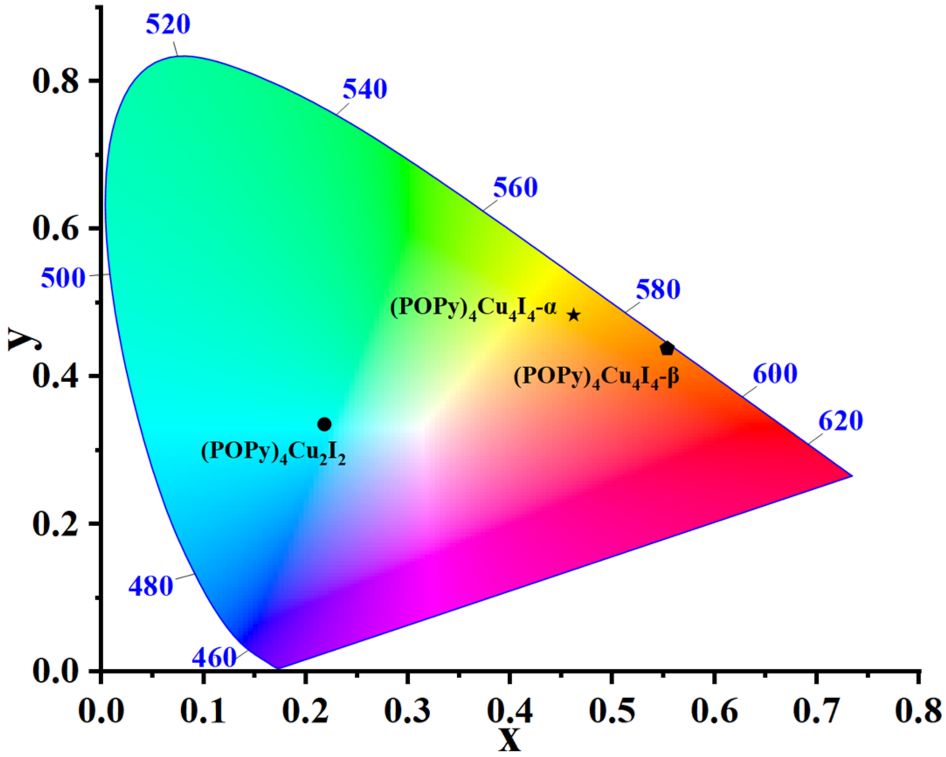

Fig. S8. CIE chromaticity diagram of $(POPy)_4Cu_2I_2$, $(POPy)_4Cu_4I_4$-α and $(POPy)_4Cu_4I_4$-β.

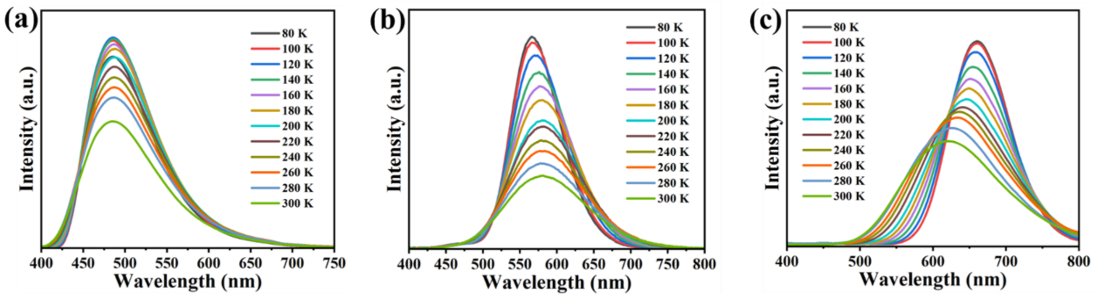

Fig. S9. Temperature-dependent PL emission spectra of (a) $(POPy)_4Cu_2I_2$, (b) $(POPy)_4Cu_4I_4$-α and (c) $(POPy)_4Cu_4I_4$-β.

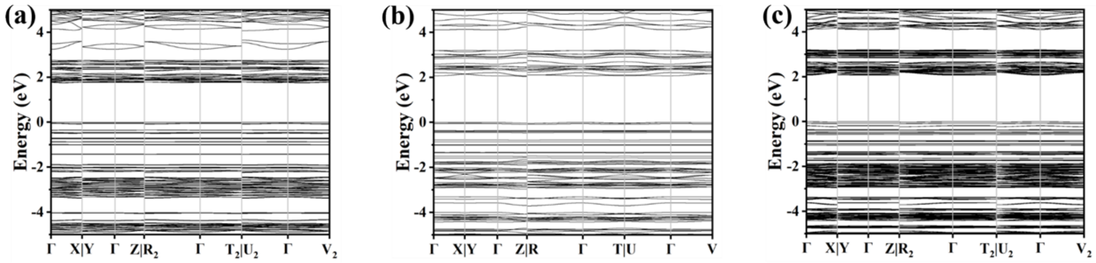

Fig. S10. Electronic band structures of (a) $(POPy)_4Cu_2I_2$, (b) $(POPy)_4Cu_4I_4$-α and (c) $(POPy)_4Cu_4I_4$-β.

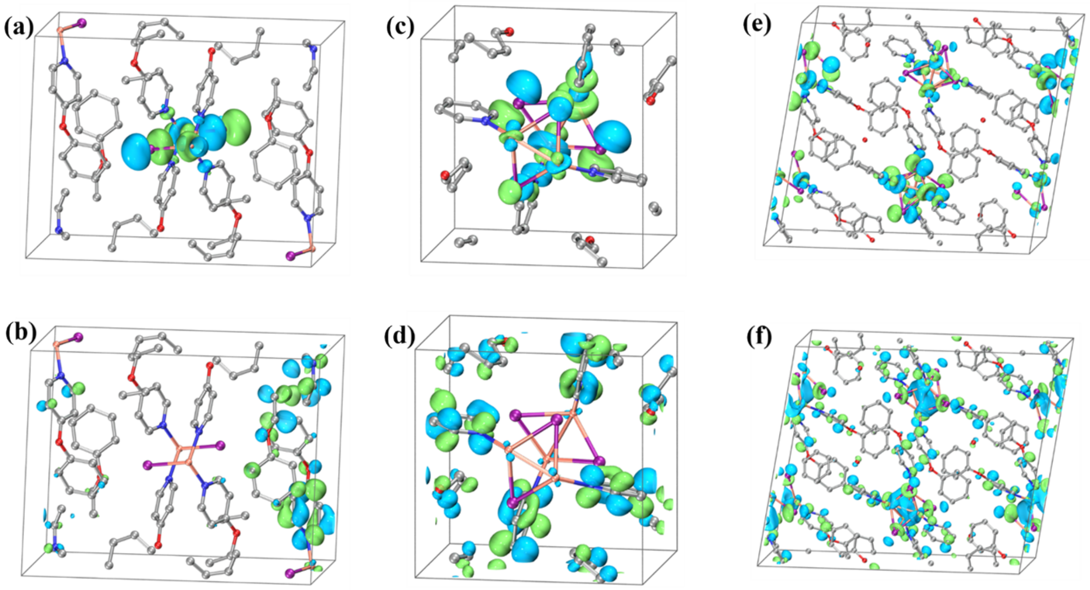

Fig. S11. Partial charge density of the (a) HOMO and (b) LUMO states for $(POPy)_4Cu_2I_2$. Partial charge density of the (c) HOMO and (d) LUMO states for $(POPy)_4Cu_4I_4$-α. Partial charge density of the (e) HOMO and (f) LUMO states for $(POPy)_4Cu_4I_4$-β.

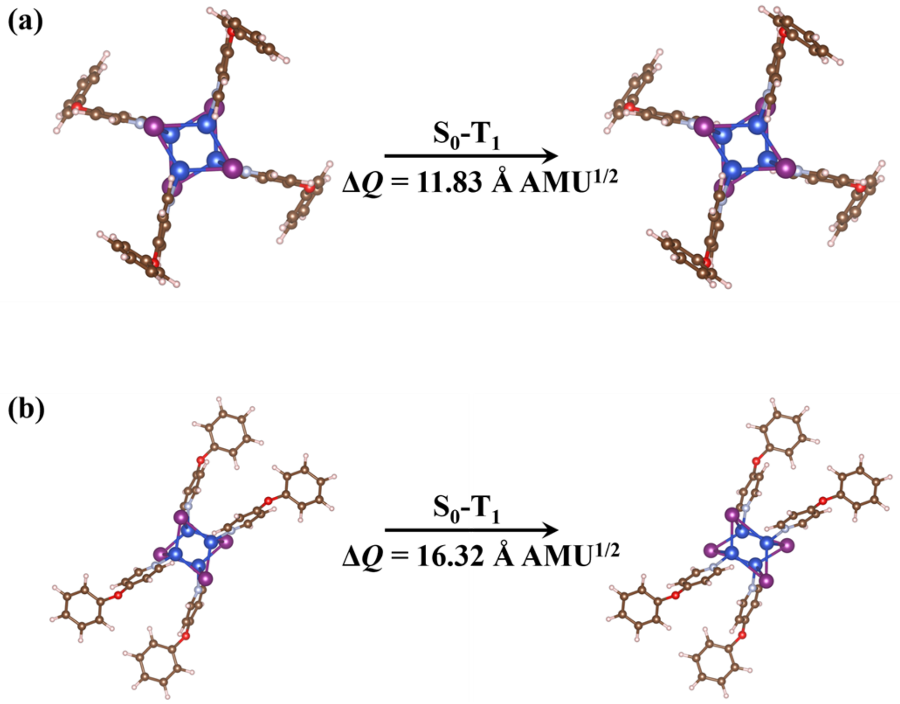

Fig. S12. (a) Excited-state structural distortion of $(POPy)_4Cu_4I_4$-α in the T_1 state compared with S_0. (b) Excited-state structural distortion of $(POPy)_4Cu_4I_4$-β in the T_1 state compared with S_0.

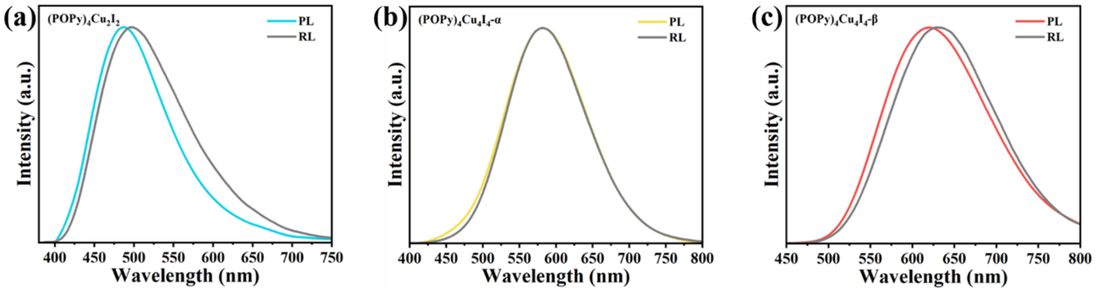

Fig. S13. Comparison of the PL and RL spectra of (a) $(POPy)_4Cu_2I_2$, (b) $(POPy)_4Cu_4I_4$-α and (c) $(POPy)_4Cu_4I_4$-β.

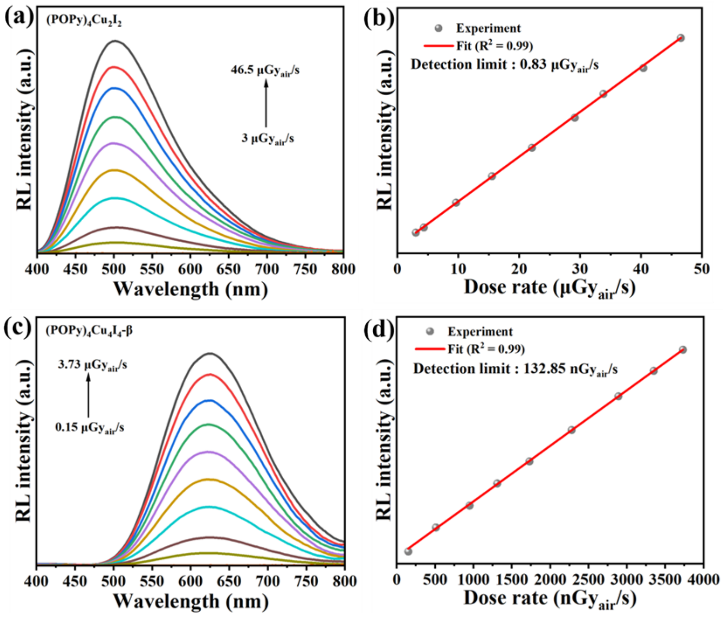

Fig. S14. (a) RL intensity spectra of $(POPy)_4Cu_2I_2$ under different X-ray dose rates ranging from 3 to 46.5 μGy_{air} S^{-1}. (b) The linear dependence of $(POPy)_4Cu_2I_2$ under different X-ray dose rate irradiation. (c) RL intensity spectra of $(POPy)_4Cu_4I_4$-β under different X-ray dose rates ranging from 0.15 to 3.73 μGy_{air} S^{-1}. (d) The linear dependence of $(POPy)_4Cu_4I_4$-β under different X-ray dose rate irradiation.

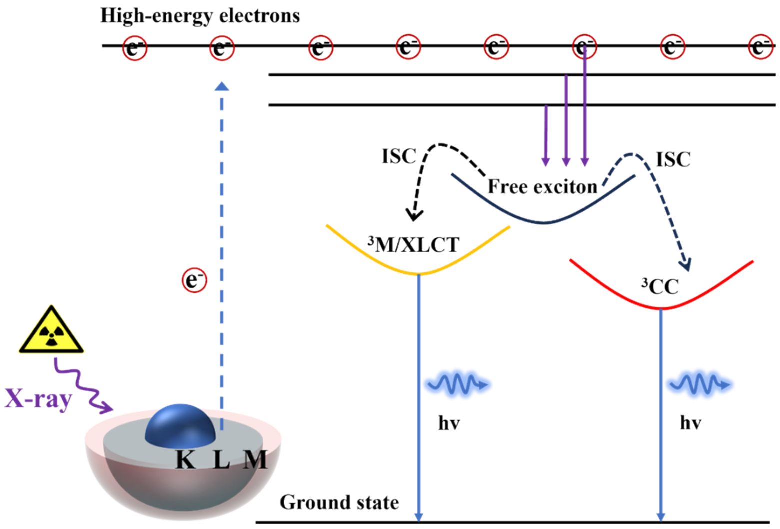

Fig. S15. Proposed radioluminescence mechanism.

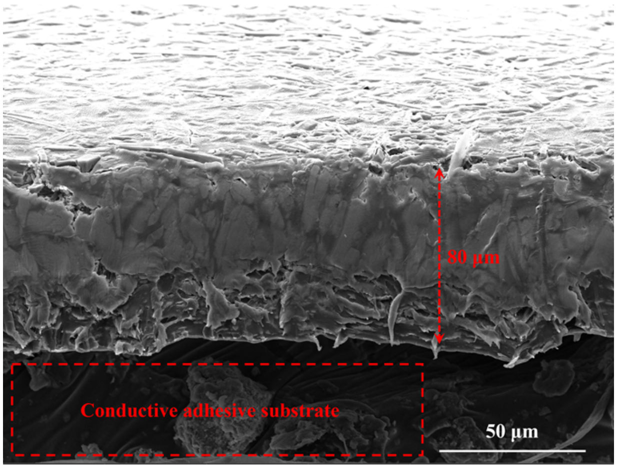

Fig. S16. Cross-sectional SEM images of $(POPy)_4Cu_4I_4$-α microcrystal-based film.

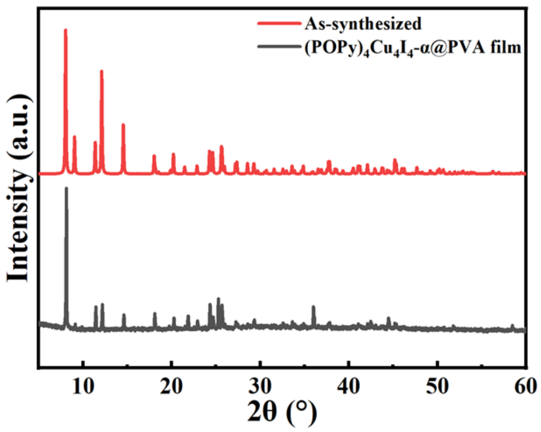

Fig. S17. The PXRD pattern of $(POPy)_4Cu_4I_4$-α@PVA film and as-synthesized sample.

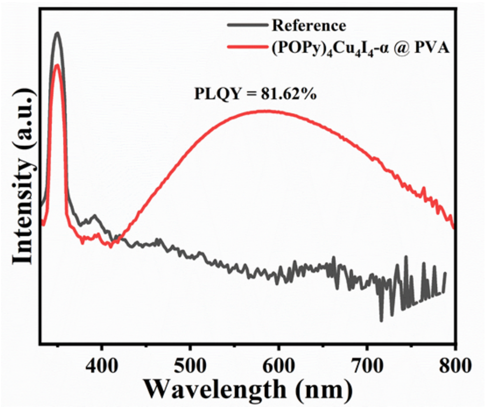

Fig. S18. The PLQY spectra of $(POPy)_4Cu_4I_4$-α@PVA film.

Table S1. Crystal data and structure refinement for $(POPy)_4Cu_2I_2$, $(POPy)_4Cu_4I_4$-α and $(POPy)_4Cu_4I_4$-β.

	**$(POPy)_4Cu_2I_2$**	**$(POPy)_4Cu_4I_4$-α**	**$(POPy)_4Cu_4I_4$-β**
**CCDC**	2391040	2391041	2391042
**Empirical formula**	$Cu_2I_2(C_{11}H_9NO)_4$	$Cu_4I_4(C_{11}H_9NO)_4$	$Cu_4I_4(C_{11}H_9NO)_4$
**Formula weight**	1065.65	1446.53	1446.53
**Temperature/K**	293.77	292.26	298.99
**Crystal system**	monoclinic	tetragonal	monoclinic
**Space group**	$P2_1/n$	P-4	C2/c
**a/Å**	8.804(2)	10.993(2)	25.128(3)
**b/Å**	18.111(4)	10.993(2)	9.0436(10)
**c/Å**	13.654(3)	9.771(2)	21.557(2)
**α/°**	90	90	90
**β/°**	105.923(6)	90	101.525(5)
**γ/°**	90	90	90
**Volume/Å^3**	2093.5(8)	1180.8(6)	4799.9(10)
**Z**	2	1	4
**ρ_{calc}g/cm^3**	1.690	2.034	2.002
**μ/mm^{-1}**	2.537	4.436	4.365
**F(000)**	1048.0	688.0	2752.0
**Index ranges**	$-8 \leq h \leq 10$, $-21 \leq k \leq 21$, $-16 \leq l \leq 16$	$-16 \leq h \leq 14$, $-15 \leq k \leq 17$, $-15 \leq l \leq 15$	$-31 \leq h \leq 20$, $-11 \leq k \leq 11$, $-26 \leq l \leq 26$
**Reflections collected**	13480	13369	17585
**Independent reflections**	3710	4717	4908
**Data/restraints/parameters**	3710/0/254	4717/0/137	4908/36/260
**Goodness-of-fit on F^2**	1.094	1.131	1.019
**Final R indexes [I>=2σ (I)]**	R_1 = 0.0297, wR_2 = 0.0526	R_1 = 0.0628, wR_2 = 0.0682	R_1 = 0.0390, wR_2 = 0.0794
**Final R indexes [all data]**	R_1 = 0.0447, wR_2 = 0.0579	R_1 = 0.1310, wR_2 = 0.0791	R_1 = 0.0628, wR_2 = 0.0897
**Largest diff. peak/hole / e Å^{-3}**	0.39/-0.48	0.73/-0.73	0.71/-0.77

$R_1 = \sum||F_o|-|F_c||\sum/|F_o|$. $wR_2 = [\sum w(F_o^2-F_c^2)^2/\sum w(F_o^2)^2]^{1/2}$

Table S2. Cu-Cu bond lengths (Å) in the three compounds.

Compounds	Cu_2I_2	Cu_4I_4-α	Cu_4I_4-β
**Cu1-Cu1[1]**	2.764(10)		
**Cu1-Cu1[1]**		2.654(16)	
**Cu1-Cu1[2]**		2.704(2)	
**Cu1-Cu1[3]**		2.654(16)	
**Cu1-Cu1[1]**			2.714(14)
**Cu1-Cu2[1]**			2.643(11)
**Cu1-Cu2**			2.669(10)
**Cu2-Cu2[1]**			2.789(14)
**Average**		2.671	2.704

Table S3. Dihedral angles of the benzene ring plane and the pyridine ring plane in the three compounds.

	Angles (°)	
**$(POPy)_4Cu_2I_2$**	67.73	82.51
**$(POPy)_4Cu_4I_4$-α**	65.67	
**$(POPy)_4Cu_4I_4$-β**	70.30	87.15

Table S4. Selected bond lengths (Å) of Cu_4I_4-α and Cu_4I_4-β.

**Cu_4I_4-α**		**Cu_4I_4-β**	
Cu-I[1]	2.6729(12)	Cu1-I1	2.6887(9)
Cu-I[2]	2.6734(13)	Cu1-I2	2.6405(8)
Cu-I[3]	2.7518(13)	Cu1-I2[1]	2.7827(9)
Cu-N	2.031(6)	Cu1-N1	2.030(4)
		Cu2-I1	2.8200(9)
		Cu2-I1[1]	2.6134(8)
		Cu2-I2	2.6867(9)
		Cu2-N2	2.018(4)

Table S5. Selected bond angles (°) of Cu_4I_4-α and Cu_4I_4-β.

**Cu_4I_4-α**		**Cu_4I_4-β**	
I^1-Cu-N	97.80(18)	N1-Cu1-I1	108.57(13)
I^1-Cu-I^2	112.45(4)	N1-Cu1-I2	97.43(13)
I^1-Cu-I^3	112.31(4)	N1-Cu1-$I2^1$	112.37(13)
I^2-Cu-N	107.97(18)	I1-Cu1-I2	119.57(3)
I^2-Cu-I^3	115.02(4)	I1-Cu1-$I2^1$	109.53(3)
I^3-Cu-N	109.81(19)	I2-Cu1-$I2^1$	108.92(3)
		N2-Cu2-I1	116.35(13)
		N2-Cu2-$I1^1$	93.63(14)
		N2-Cu2-I2	106.59(13)
		I2-Cu2-I1	118.31(3)
		I2-Cu2-$I1^1$	110.42(3)
		I1-Cu2-$Cu1^1$	110.81(3)

Table S6. Degree of distortion present in Cu_4I_4-α and Cu_4I_4-β.

	**Cu_4I_4-α**	**Cu1 of Cu_4I_4-β**	**Cu2 of Cu_4I_4-β**
D_{tet}	9.898×10^{-2}	9.968×10^{-2}	1.019×10^{-1}
σ^2	10.023	22.307	27.295

[1] J. V. Vondele, M. Krack, F. Mohamed, M. Parrinello, T. Chassaing and J. Hutter, *Comput. Phys. Commun.*, 2005, **167**, 103.

[2] T. D. Kuhne,M. Lannuzzi, M. D. Ben, V. V. Rybkin, P. Seewald, F. Stein, T. Laino, R. Z. Khaliullin, O. Schutt, F. Schiffmann, D. Golze, J. Wilhelm, S. Chulkov, M. H. Bani, V. Weber, U. Vorstnik, J. V. Vondele, M. Krack and J. Hutter *J. Chem. Phys.*, 2020, **152**, 194103.

[3] J. P. Perdew, K. Burke and M. Ernzerhof, *Phys. Rev. Lett.*, 1996, **77**, 3865.

[4] S. Grimme, S. Ehrlich and L. Goerigk, *J. Comput. Chem.* 2011, **32**, 1456.

[5] J. Heyd, G. E. Scuseria and M. Ernzerhof, *J. Chem. Phys.*, 2006, **124**, 219906.

[6] J. V. Vondele and J. Hutter, *J. Chem. Phys.*, 2007, **127**, 114105.

[7] W. Humphrey, A. Dalke and K. Schulten, *J. Mol. Graph.*, 1996, **14**, 33.

[8] T. Lu and F. Chen, *J. Compu.t Chem.*, 2012, **33**, 580.
